# Molecular mechanism of ectopic lipid accumulation induced by methylglyoxal *via* activation of the NRF2/PI3K/AKT pathway implicates renal lipotoxicity caused by diabetes mellitus

**DOI:** 10.1371/journal.pone.0306575

**Published:** 2024-10-16

**Authors:** Chiung Chi Peng, Eugene Chang Yu Chen, Chang-Rong Chen, Charng-Cherng Chyau, Kuan-Chou Chen, Robert Y. Peng

**Affiliations:** 1 Graduate Institute of Clinical Medicine, College of Medicine, Taipei Medical University, Taipei, Taiwan; 2 MD Program, Medical College of Wisconsin, Milwaukee, Wisconsin, United States of America; 3 Department of Biotechnology, College of Medical and Health Care, Hungkuang University, Shalu District, Taichung, Taiwan; 4 Department of Urology, Taipei Medical University Shuang-Ho Hospital, Zhong-He District, New Taipei City, Taiwan; 5 TMU-Research Center of Urology and Kidney, Taipei Medical University, Taipei, Taiwan; Max Delbruck Centrum fur Molekulare Medizin Berlin Buch, GERMANY

## Abstract

Patients with chronic kidney disease (CKD) have a high incidence of dyslipidemia comprising high triglyceride (TG) and low high-density lipoprotein (HDL)-cholesterol levels. An abnormal increase of TGs within cells can lead to intracellular lipid accumulation. In addition to dyslipidemia, hyperglycemia in diabetes may elicit ectopic lipid deposition in non-adipose tissues. Hyperglycemia increases intracellular levels of methylglyoxal (MG) leading to cellular dysfunction. A deficit of glyoxalase I (GLO1) contributes to dicarbonyl stress. Whether dicarbonyl stress induced by MG causes renal lipotoxicity through alteration of lipid metabolism signaling is still unknown. In this study, mice with high fat diet-induced diabetes were used to investigate the renal pathology induced by MG. NRK52E cells treated with MG were further used *in vitro* to delineate the involvement of lipogenic signaling. After treatment with MG for 12 weeks, plasma TG levels, renal fatty changes, and tubular injuries were aggravated in diabetic mice. In NRK52E cells, MG activated the nuclear factor erythroid 2-related factor 2 (Nrf2)/phosphatidylinositol 3-kinase (PI3K)/protein kinase B (AKT) and sterol regulatory element-binding protein 1 (SREBP1), resulting in stimulation of fatty acid synthase. The intracellular accumulation of lipid droplets was mainly contributed by TGs, which increased the oxidative stress accompanied by high Nrf2 expression. In addition, MG time-dependently activated cyclin D, cyclin-dependent kinase 4 (CDK4), and cleaved caspase-3, evidencing that G_0_/G_1_ arrest was associated with apoptosis of NRK52E cells. In conclusion, our studies revealed the mechanism of lipotoxicity caused by MG. The target of such dicarbonyl stress may become a promising therapy for diabetic CKD.

## 1. Introduction

Dyslipidemia is the leading cause of morbidity and mortality in CKD and type 2 diabetic mellitus (DM; T2DM) patients [1]. Ectopic lipid accumulation mainly manifests as dysfunctional lipid signaling and insulin resistance responses in non-adipose tissues, including the myocardium, pancreas, skeletal muscle, liver, and kidneys [2]. Lipid accumulation in kidney tissues may cause renal lipotoxicity and can lead to acute kidney injury, which further turns into CKD and renal fibrosis [3]. Pathologically, lipotoxicity can be initiated by several causes, including dysfunctional glucose transporter 4 (GLUT4) receptor signaling, a dysfunctional lipid storage capacity, an increase in non-esterified fatty acid (FA), defective insulin signaling, and dysfunctional gluconeogenesis in active lipogenesis. In addition, there is an excessive uptake of extracellular FAs caused by an overactive cluster of differentiation 36 (CD36) [2] and FA-binding proteins (FABPs) [4]. FA metabolism is regulated by AMP-activated protein kinase (AMPK), peroxisome proliferator-activated receptor-γ (PPARγ) coactivator-1α (PGC-1α), PPARα, carbohydrate response element-binding protein (ChREBP), and sterol regulatory element-binding protein (SREBP) [2], where SREBP mediates the progression of CKD, including lipid-dependent and lipid-independent pathways [5]. Upregulation of nuclear SREBP1c enhances expressions of lipogenic genes, the synthesis of FAs, and the accumulation of TGs [6]. Notably, SREBP-1 and fatty acid synthase (FAS) are upregulated in DM animal models [7].

Hyperglycemia mechanistically plays an essential role in diabetic kidney diseases, in which the polyol pathway, advanced glycation end products (AGE), oxidative stress, proinflammatory cytokines, profibrotic growth factors, etc., are all involved [8]. MG is a reactive-dicarbonyl that is thought to contribute to the development of diabetes as a precursor for AGE, impairing vascular function, inducing mitochondrial dysfunction, and stimulating the hexosamine pathway [9]. GLO1 is the rate-limiting enzyme that participates in MG detoxification. Dicarbonyl stress, mainly caused by MG, is the abnormal accumulation of reactive dicarbonyl metabolites leading to cell and tissue dysfunction [10], in particular, leading to chronic renal disease and cardiovascular mortality in individuals with T2DM [11].

Several studies revealed that the regulatory antioxidant response element in the *Glo1* gene is inducible by activation of the cytoprotective transcription factor, nuclear Nrf2, which controls both basal and inducible expressions of *Glo1* in response to dicarbonyl stress [9, 12]. Nrf2 is one of the intracellular redox mediators in response to oxidative stress. Previously, the PI3K/protein kinase B (AKT) pathway was shown to play an important role in modulating Nrf2 signaling for cytoprotection [13]. It is well established that the PI3K/AKT pathway is a main regulator of diverse cellular processes involved in cell survival, proliferation, metabolism, and apoptosis. PI3K/AKT was suggested to regulate glucose metabolism through Forkhead box protein O1 (FoxO1) and glycogen synthase kinase 3 (GSK-3) and lipid metabolism through mammalian target of rapamycin complex 1 (mTORC1) and SREBP in obesity and T2DM [14].

MG is directly toxic to tissues, but whether an increase of plasma MG levels elicits renal lipotoxicity is still unclear. To attain an explicit approach, MG-related lipotoxicity was validated in a diabetic animal model. We then used an NRK52E cell model to investigate the damaging effect of MG on the energy sensing system, on anti-dicarbonyl stress by Nrf2, and on regulatory molecules associated with FA biosynthesis and transport.

## 2. Materials and methods

### 2.1. Chemicals

An MG solution was purchased from Sigma-Aldrich (St. Louis, MO, USA). Nile red was supplied by Santa Cruz Biotechnology (Santa Cruz, CA, USA). The PRO-PREP Protein Extraction Solution was provided by iNtRON Biotech (Kyungki-Do, Korea).

BrBzGCp2, Mangiferin, Resveratrol and Hesperetin were purchased from MedChemExpress LLC. (Monmouth Junction, NJ, USA). All other chemicals were purchased from BioShop Canada (Burlington, Ontario, Canada) unless otherwise noted.

### 2.2. Animal experiment

All animal experiments were approved by the Institutional Animal Care and Use Committee (IACUC) at Taipei Medical University. In brief, C57BL/6 mice aged 6 weeks, with body weight (BW) ranging 20~22 g, purchased from BioLASCO (Taipei, Taiwan), were admitted to the animal room and acclimated at 26±1°C and 65% relative humidity, with a light/dark cycle controlled to 12/12 h, and were fed regular chow (Oriental Yeast, Tokyo, Japan) for 1 week. In the second week, their BWs were measured, then the mice were divided into three groups: Group A, normal diet control; Group B, a high-fat diet (HFD); and Group C, HFD diet plus MG (30 mg/kg, i.p.). (n = 8 mice/group). BWs were weighed every week until week 17. One day before euthanized, urine was collected (each mouse was placed in a metabolic cage at 18:00 and collected at 10:00 the next day). At week 17, all animals were euthanized under isoflurane anesthesia. The blood, urine and kidneys were sampled and analyzed. Immunohistochemical staining of anti-CD36 (Taiclone, Taipei, Taiwan) was performed according to our previous study [3].

### 2.3. Source and culture of the NRK52E cell line

The NRK52E, purchased from the Bioresource Collection and Research Center (BCRC) (Hsinchu, Taiwan), is a rat renal proximal tubular cell line. Cells were cultured in Dulbecco’s modified Eagle medium (DMEM) with L-glutamine (4 mM), 1.5 g/L sodium bicarbonate, 4.5 g/L glucose, and 5% bovine calf serum at 37°C under 5% CO_2_. A subcultivation ratio of 1:3 to 1:4 was recommended.

### 2.4. MTT cell viability test

NRK52E cells were seeded onto a 24 well-plate at a density of 2.5×10^4^ cells/well and incubated at 37°C under a 5% CO_2_ for 24 h or until 90% confluent cell adhesion. Cells were divided according to the experimental groups. The medium was aspirated off, MG (0~800 μM) or Glo 1 inducer/inhibitor were applied, and incubation was continued for 0~48 h. The medium was aspirated away, 400 μL of an MTT solution (0.5 mg/mL) was added, and the culture was re-incubated for 3 h. The medium was again aspirated off, and 100 μL DMSO was added to dissolve the purple formazan crystals produced *via* reduction of MTT by dehydrogenase. The optical density (OD) was read against the control at 595 nm with an enzyme-linked immunosorbent assay (ELISA) reader. A higher OD indicated higher viable cell viability. The viability percentage was calculated from Eq 1:

$$\%\;Viability\;=\;\left(\frac{{OD}_{sample}}{{OD}_{control}}\right)\times 100\%.$$ 1

Data obtained from triplicate experiments were treated statistically and expressed as the mean±standard deviation (SD) (*n* = 3). The significance level was set to *p*<0.05 with respect to the control.

### 2.5. Cell cycle flow cytometry

NRK52E cells were seeded onto 10-cm dishes at a density of 2.5×10^4^ cells until 70% confluent. The designed dosage of drugs was applied and examined at different preset time points. Cells were rinsed once with ice-cooled phosphate-buffered saline (PBS), and 1 mL 0.25% trypsin and a 0.02% EDTA solution were added to disperse cells completely. Cells were rinsed twice with ice-cooled PBS. Cells were completely dispersed with the aid of the residual liquid on a vibrator as it slowly stirred, and at the same time, 2 mL of ethanol (70%) was slowly added to ensure the complete dispersion of cells. Cells were refrigerated at -20°C for at least 2 h until well-fixed. Immediately before staining, cells were rinsed twice with ice-cooled PBS and centrifuged at 1500 ×*g* for 5 min to eliminate the supernatant. Cells were dispersed with the aid of the residual fluid. The propidium iodide (PI) staining agent was added to cells, mixed well, and left to stand for 1 h while avoiding direct light. Cells were vortexed for complete dispersion immediately before being subjected to flow cytometry.

### 2.6. Analysis of cell apoptosis

NRK52E cells were seeded on 10-cm dishes at a density of 2.5×10^4^ cells until 70% confluent. The designed dosage of drugs was applied and examined at different preset time points. The medium was aspirated off. Cells were rinsed once with PBS, and 1~2 mL trypsin (0.25%) and an EDTA (0.02%) solution were added to disperse cells completely. An additional culture medium was added to terminate the reaction. Medium (100 μL) containing serum was added and completely dispersed. To the cell suspension, 100 μL Muse™ Annexin V & Dead Cell Reagent (Merck Millipore, Burlington, MA, USA) was added and left to stand at ambient temperature for 20 min while avoiding direct sunlight. Cells were vortexed for complete dispersion immediately before being subjected to flow cytometry on a Muse^®^ Cell Analyzer (Luminex, Austin, TX, USA).

### 2.7. Extraction of cytoplasmic and nuclear proteins

NRK52E cells were seeded onto six-well plates at a density of 1.5×10^6^ cells and treated with the test drugs as indicated. Tested cells were collected at indicated time points. The medium was removed, and pellets were rinsed once with PBS. Cells were rinsed once with PBS, and 1 mL 0.25% trypsin and a 0.02% EDTA solution were added to completely detach cells and centrifuged at 1500 ×*g* for 5 min, and the supernatant was discarded. Cells were treated as instructed by the manufacturer to obtain cytoplasmic proteins and nucleoproteins (Nuclear & Cytoplasmic Extraction Kit, G-Biosciences, St. Louis, MO, USA). The supernatant was transferred to new Eppendorf flasks and stored at -80°C for use.

### 2.8. Sodium dodecyl sulfate polyacrylamide gel electrophoresis (SDS-PAGE) electrophoresis and Western blotting

Total protein 30 mg was heated to 100°C for 10 min to denature the proteins, and then directly transferred to a sample well of 10% SDS-PAGE and subjected to 65 mV to carry out stacking gel electrophoresis. Then proteins were transferred onto the polyvinylidene difluoride (PVDF) membranes. Membranes were then incubated for 16 h at 4°C with anti-cyclin D, anti-Glo-1 (Santa Cruz Biotechnology, Dallas, TX, USA), anti-cleaved caspase 3, anti-phosphorylated (p)-AMPK, anti-AMPK, anti-AKT, anti-acetyl CoA carboxylase, anti-FAS, anti-p-PI3K, anti-PI3K, anti-p21 (Cell Signaling Technology, Danvers, MA, USA), anti-p-AKT (Epitomics, Boston, MA, USA), anti-3-hydroxy-3-methyl-glutaryl-coenzyme A reductase (HMGCR) (BioVision, Boston, MA, USA), anti-FA transport protein 2 (FATP2) (Bioss, Woburn, MA, USA), anti-cluster of differentiation 36 (CD36) (Taiclone Biotech, Taipei, Taiwan), anti-cyclin-dependent kinase 4 (CDK4), anti-SREBP1, anti-SREBP2, anti-NRF2, anti-histone deacetylase (HDAC), anti-peroxisome proliferator-activated receptor-α (PPARα), anti-PPARγ (GeneTex, Irvine, CA, USA), and anti-β-actin (Novus Biologicals, Centennial, CO, USA). The secondary antibody tagged with horseradish peroxidase (HRP; 1:1000) was added, left to react for 1 h at ambient temperature, and then rinsed trice with TBST for 10 min. An electrochemiluminescence (ECL) agent (Merck Millipore, Billerica, MA, USA) was applied to excite the chemiluminescence emission from proteins on the PVDF membrane. A blot image was taken and quantified with ImageJ software (National Institutes of Health, USA).

### 2.9. Assay for intracellular glyoxalase activity

NRK52E cells were cultured in 10-cm dishes until 70% confluent. The designed dosage of drugs was applied and examined at different set time points. The medium was aspirated off. Cells were rinsed once with PBS. Cells (2.5×10^4^) were harvested and suspended in 300 μL of an analytical solution, and glyoxalase I activity was measured using a Glyoxalase I Activity Assay Kit (Biovision, San Francisco, CA, USA). The activity was expressed in units/mg protein.

### 2.10. Determination of intracellular oxidative stress

NRK52E cells were cultured in 10-cm dishes until 70% confluent. The cells being treated with MG (0–700 μM) were incubated for 0.5, 1, 2, and 4 h. Hydrogen peroxide was used as the positive control. Muse^®^ Oxidative Stress Kit (Merck Millipore, Burlington, MA, USA) was assigned to determine intracellular oxidative stress with a Muse^®^ Cell Analyzer (Luminex).

### 2.11. 3D Cell Explorer analysis of lipid droplets in NRK52E cells

NRK52E cells were cultured for 24 h in 35-mm μ-Dishes (ibidi GmbH, Gräfelfing, Germany) (a 35-mm micro-dish with an ibidi Polymer Coverslip bottom for high-end microscopy). Cells treated with 500 μM MG were further incubated for 24 h. The medium in the sample was aspirated off and replaced with colorless medium to avoid any interference from light diffraction. The interior of cells was scanned with Nanolive 3D Cell Explorer (Nanolive, Tolochenaz, Switzerland). In principle, taking advantage of Nanolive imaging and analysis platforms, 3D Cell Explorer measures the intracellular density of live cells by analyzing differential 3D diffraction characteristics of live cells, and the distribution of lipid droplets (or target organelles) in live cells can be easily accessed by illustrating target organelles.

### 2.12. Confocal microscopy of Nile red staining

After being sterilized, coverslips were placed onto 24-well plates. Cells were seeded into each well at a density 2.5×10^4^ cells/well and left to stand for 24 h. To each well, MG (500 μM) was added. Cells were further cultured for 48 h, the medium was aspirated off, and cells were rinsed twice with PBS. Paraformaldehyde (4%) was added to fix cells at ambient temperature for 1 h, and then they were rinsed twice with PBS. Nile red (2.5 μg/mL in PBS) (Santa Cruz Biotechnology, Dallas, TX, USA) was added and left to stand at ambient temperature for 15 min while avoiding direct sunlight. Excess stain was removed with five washes of cold PBS (137 mM NaCl, 2.7 mM KCl, 10 mM Na_2_HPO_4_, and 1.8 mM KH_2_PO_4_, at pH 7.4). After being drained, sealed with Fluoromount-G™ Mounting Medium with 4’,6-diamidino-2-phenylindole (DAPI) (Thermo Fisher Scientific, Waltham, MA USA), air dried at ambient temperature, and stored at 4°C, the finished slips were observed within 1 week with an inverted microscope and a laser confocal microscope (magnification 200×), and photos were taken at the same time. ImagePro software was used to analyze and quantify the area under Nile red-stained curves. Data were treated statistically, and results are expressed as the mean±standard deviation (SD) (*n* = 3).

### 2.13. Nile red flow cytometric analysis of contents of intracellular lipids in NRK52E cells

NRK52E cells were cultured in 10-cm dishes until 70% confluent. The designed dosage of drugs (0, 300, 500, 700 μM MG) was applied, cultured for 48 h, and examined at different set time points. The medium was aspirated off, cells were rinsed once with PBS, and 1~2 mL 0.25% trypsin and a 0.02% EDTA solution were added to completely disperse cells. An additional culture medium was added to terminate the reaction. The supernatant was aspirated off. Cells were rinsed twice with ice-cooled PBS. After the greatest proportion of the supernatant had been aspirated off, cells were completely dispersed in the residual fluid. Nile red agent (Nile red 1 μg/mL in PBS) was added, and the mixture was left to stand at ambient temperature for 15 min while avoiding direct sunlight. Cells were vortexed to completely disperse cells immediately before being subjected to a flow cytometric analysis. The fluorescence intensity at 2000 cells/event was measured with a Muse^®^ Cell Analyzer (Luminex).

### 2.14. Determination of the intracellular TG level in NRK52E cells

NRK52E cells were cultured in 10-cm dishes until 70% confluent. The designed dosage of MG (500 μM) was applied and cultured for 48 h. The medium was aspirated off, and cells were disrupted on ice with ultrasonication which was operated in a mode of 5 s of operation/20 s stopped for each cycle. The suspension was centrifuged at 10^4^ ⋅*g* for 10 min. The TG contents were measured with a Triglyceride Colorimetric Assay Kit (cat. no. 10010303, Cayman Chemical, Ann Arbor, MI, USA). TG contents are expressed as the mean±SD (mg/dL) from quadruplicate experiments.

### 2.15. Nrf2 transcription factor assay

NRK52E cells were cultured in 10-cm dishes until 70% confluent and incubated for MG 500 μM at 30 min, and 1h. The medium was aspirated off. Cells were rinsed once with PBS. Nuclear extracts were harvested and suspended in 300 μL of an analytical solution, and Nrf2 transcription factor activity was measured using a Nrf2 Transcription Factor Assay Kit (Abcam, Boston, MA, USA). The activity was measured at OD 450 nm.

### 2.16. Statistical analysis

Data obtained from triplicate experiments were statistically analyzed using GraphPad Prism 7 graphic software (GraphPad Software, Boston, MA, USA) and expressed as the mean±standard error of the mean (SEM) (*n* = 3). The variation between every two groups was assessed using a one-way analysis of variance (ANOVA) and the Bonferroni test. The significance level was set to *p*<0.05.

## 3. Results

### 3.1. MG aggravated fat deposition in renal tissues of diabetic rats

**Fig 1:**
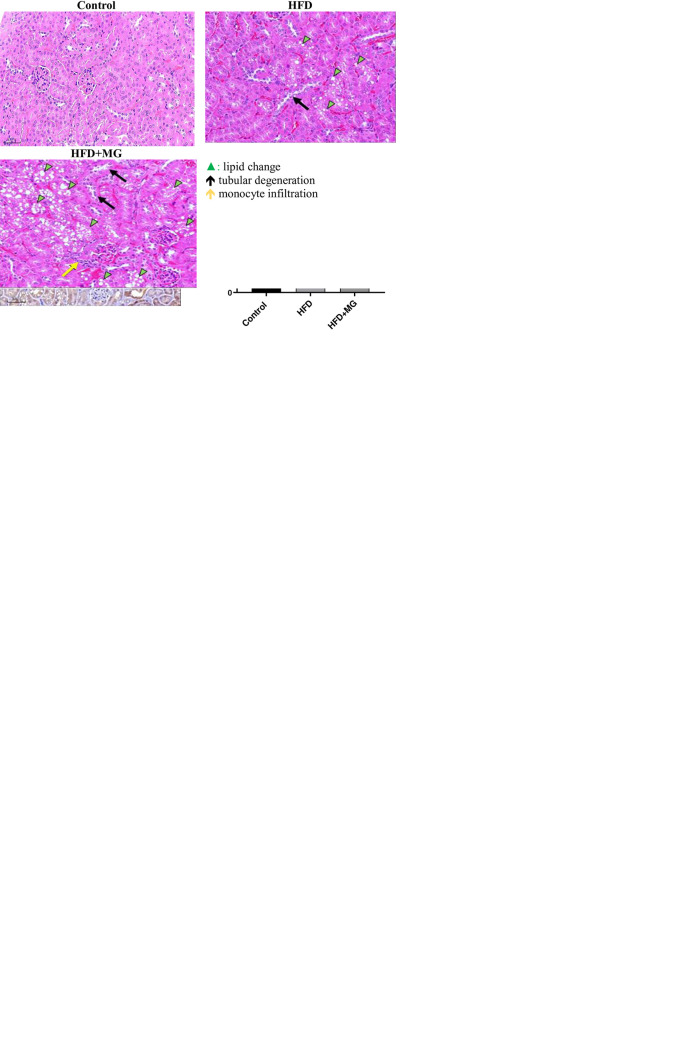
Experimental data from the diabetic mice model of control, HFD, and HFD+MG groups (*n* = 8 in each group). (a) Body weight changes during the 15-week experiment (b) Plasma sugar level (c) Plasma triglyceride levels (d) Plasma cholesterol levels (e) H&E examination of kidney tissues (f) Pathological scores of kidney fatty changes (g) Pathological scores of renal tubular degeneration (h) Pathological scores of mononuclear cell infiltration (i) The immunohistochemical staining and quantification of CD36 in the control, HFD, and HFD+MG groups. green▲, lipid change; black ↑, tubular degeneration; yellow ↑, monocyte infiltration. *vs. the control group, ^#^vs. the HFD group (^#^*p*<0.05, **p<0.01, ***p<0.001).The pathology report (no: 107–20040) was provided by National Applied Research Laboratories (Taipei, Taiwan).

BWs of the HFD-only group rapidly increased compared to those of the control group from weeks 1 to 15. BWs of the HFD+MG group increased more slowly than those of the HFD group (**Fig 1A**). Blood glucose levels had increased in the HFD and HFD+MG groups compared to the controls by week 15 (*p*<0.01) (**Fig 1B**). As shown in **Fig 1C**, total plasma TGs were significantly elevated in the HFD and HFD+MG groups compared to the control group. Moreover, TG levels in the HFD+MG were higher than those of the HFD group. Plasma cholesterol of the HFD and HFD+MG groups was significantly higher than that in the control group. However, the plasma cholesterol of the HFD+MG group was lower compared to the HFD group (**Fig 1D**). The pathological report revealed fatty changes in the kidneys of the HFD groups. The fatty change score in the HFD+MG group was much higher than that in the HFD group (**Fig 1F**). Renal injury was demonstrated by complications, including renal tubular degeneration and fat deposition in the HFD and HFD+MG groups (**Fig 1E and 1F**). In addition, greater renal lipogenesis was observed in MG-treated HFD rats (**Fig 1F**). In our previous study, increased lipid deposition by oleic acid supplement demonstrated decreased cell viability in NRK52E (Lin et al., 2019).

### 3.2. MG inhibited the proliferation and induced apoptosis in NRK52E cells

**Fig 2:**
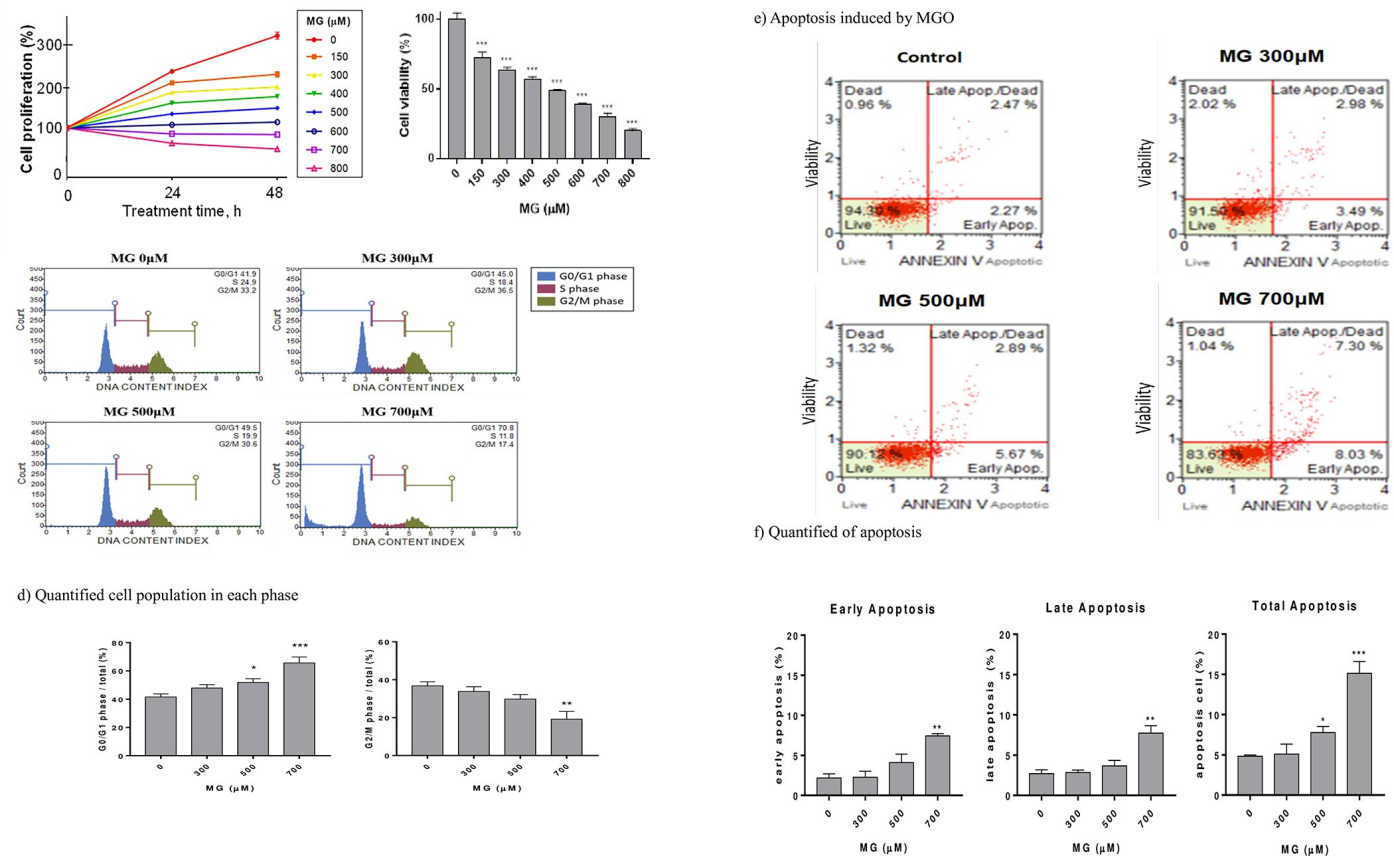
Effect of MG on the cell viability, cell cycle, and apoptosis of NRK52E cells. (a) MTT assay of NRK52E cells at 24 and 48 h after MG treatment (0~800 μM). (b) Quantification of cell viability at 48 h affected by MG compared to the control (100%). NRK52E cells were seeded onto 24-well plates at a density of 2.5×10^4^ cells/well and treated with MG (0~800 μM) for 24~48 h, after which an MTT assay was carried out. Data obtained from triplicate experiments were treated statistically and are expressed as the mean±SD (*n* = 3); asterisk superscripts indicate a significant difference with respect to the control (*** *p*<0.001). (c) Flow cytometric analysis affected by MG treatment at 48 h. (d) Quantification of the cell cycle at 48 h. The abscissa represents the propidium iodide (PI) fluorescence intensity, and the ordinate is cell counts compared to the control. (e) Apoptotic analysis of NRK52E cells by MG treatment at 48 h. (f) Quantification of the population of apoptotic cells. NRK52E cells were treated as described above with MG at 0~700 μM for 48 h. Data were statistically treated, and the results are expressed as the mean±SD (*n* = 3). Percentages of the cell cycle and apoptosis are shown. Different levels of significance are expressed as * *p*<0.05, ** *p*<0.01, and *** *p*<0.001.

MG dose- and time-dependently inhibited NRK52E cell growth and viability (**Fig 2A and 2B**). Cell growth was halted by MG treatment at 24 and 48 h (**Fig 2A**). The 50% inhibition concentration (IC_50_) of viability occurred at around 450 μM (**Fig 2B**). The cell cycle analysis showed that G_0_/G_1_ cell population dose-dependently increased from 45.0% (300 μM MG) to 49.5% (500 μM) and 70.8% (700 μM), compared to 41.9% of the control (**Fig 2C and 2D**); while the G_2_/M phase population correspondingly decreased from 36.5% and 30.6% to 17.4% at 300, 500, and 700 μM, respectively, compared to 33.2% of the control (**Fig 2C and 2D**). MG (>500 μM) stopped the cell cycle at G_0_/G_1_ phase in NRK52E cells (**Fig 2C and 2D**).

MG also induced cell apoptosis in NRK52E cells (**Fig 2E and 2F**). The population of early apoptotic cells increased from 2.27% (0 μM MG) to 3.49% (300 μM MG), and 5.67% (500 μM MG), and finally to 8.03% (700 μM MG). In contrast, correspondingly late apoptosis increased from 2.98% to 2.89% and 7.30% compared to 2.47% of the control (**Fig 2E and 2F**). MG significantly induced total apoptosis at both 500 (8.56%) and 700 μM (15.33%) in NRK52E cells (**Fig 2F**).

### 3.3. G1-phase cell cycle- and apoptosis-associated proteins in NRK52E cells were affected by MG

**Fig 3:**
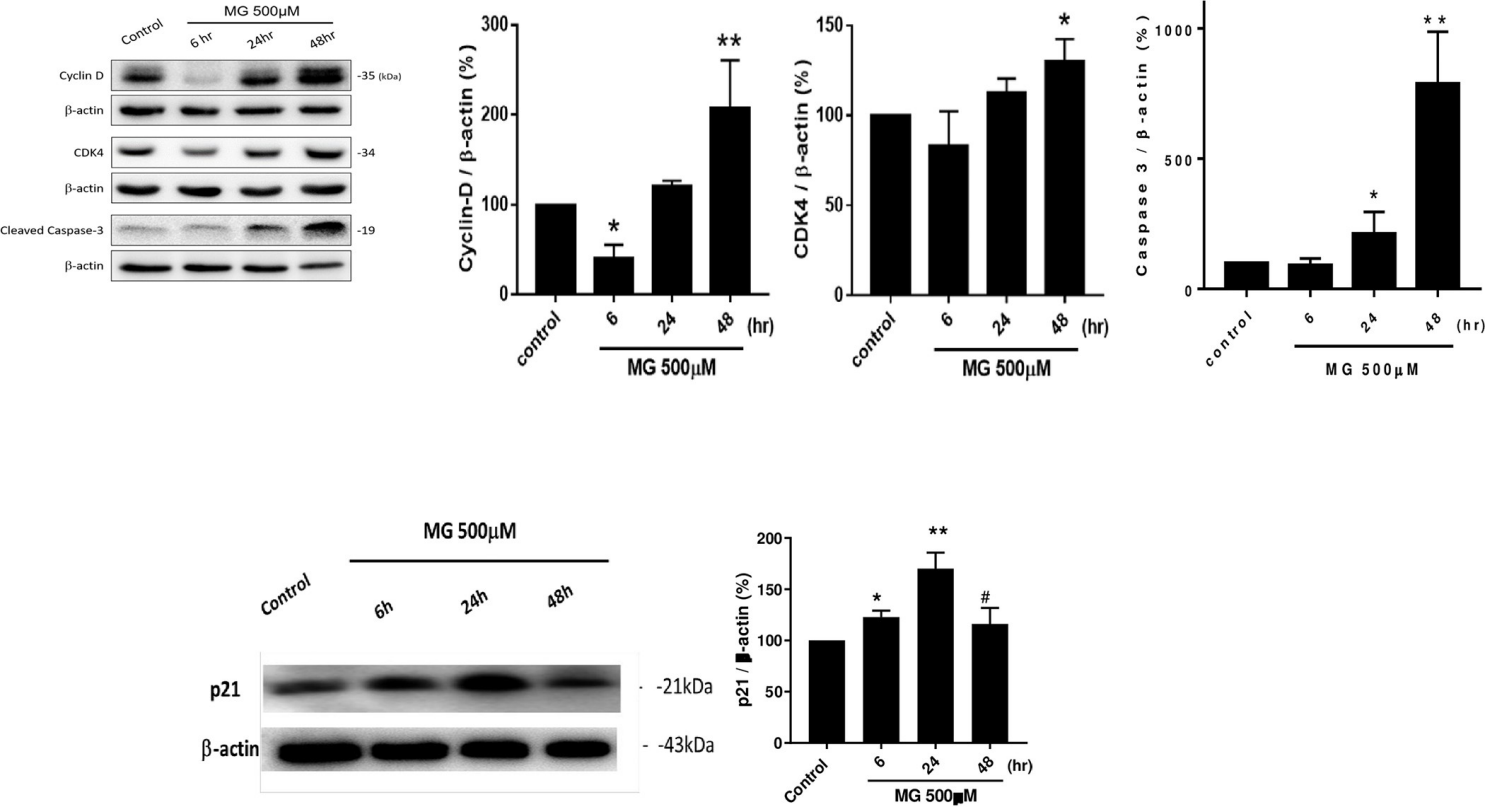
Cell cycle- and apoptosis-related proteins affected by MG. (a) Western blotting of cyclin D, CDK4, and cleaved caspase-3. β-Actin was used as an internal control. (b) Quantified bar diagrams. NRK52E cells were treated with 500 μM MG for 6, 24, and 48 h. (c) Western blotting and quantification of p21. β-Actin was used as an internal control. Proteins were collected from cell lysates and subjected to Western blotting. Data were statistically treated, and results are expressed as the mean±SD (*n* = 3). Different levels of significance are expressed as * *p*<0.05 and ** *p*<0.01 compared to the control, #p<0.05 compared to 24h.

Both cyclin D and CDK4 displayed similar expression patterns in response to 500 μM MG. Amounts of both were immediately downregulated at 6 h and then steadily upregulated to 48 h. The most prominently expressed stage for cyclin D and CDK4 occurred at 48 h, reaching 210% (*p*<0.01) and 133% (*p*<0.05), respectively, compared to the control (100%) **(Fig 3A and 3B)**. In contrast, cleaved caspase 3 expression was unaffected until 6 h but had increased to 200% by 24 h and then to 800% by 48 h (*p*<0.01) (**Fig 3A and 3B**). In parallel, p21 was increased progressively to 123% (p<0.05) and 176% (p<0.01) by 6 and 24h but decreased to 116% (p<0.05 vs. 24h) at 48h when cells treated with 500 μM MG (**Fig 3C**).

### 3.4. MG upregulated GLO-1 activity and expression in NRK52E cells

**Fig 4:**
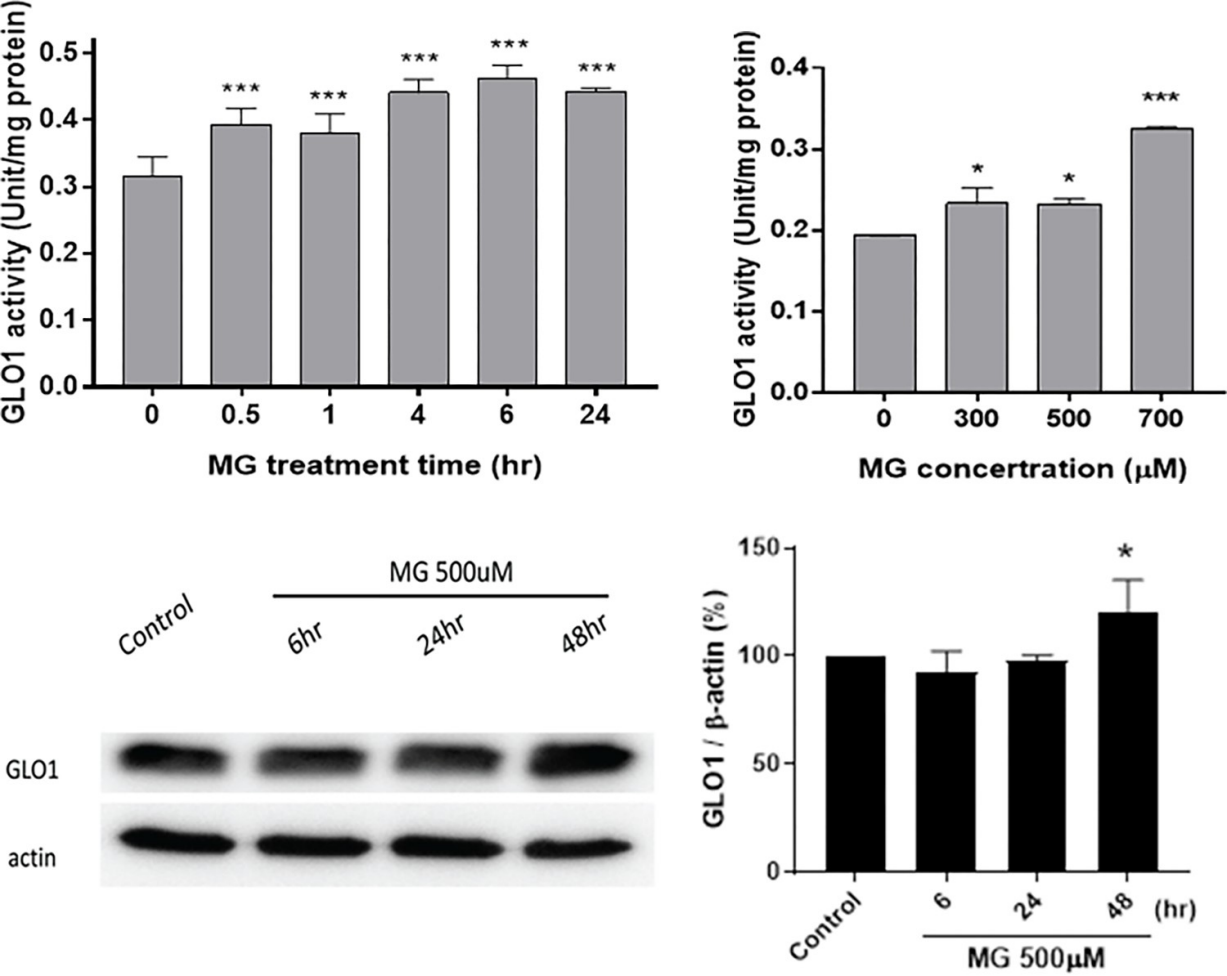
Expression of intracellular glyoxalase 1 (GLO-1) affected by MG. NRK52E cells were treated with 0~700 μM MG for 0~48 h. (a) Time-dependent stimulation of GLO-1 activity by 500 μM MG in NRK52E cells for 0~24 h. (b) GLO-1 activity in NRK52E cells affected by 300, 500, and 700 μM MG. GLO-1 activity was measured with a Glyoxalase I Activity Assay Kit (Biovision). GLO-1 activity is expressed as units/mg protein. (c) Western blotting of the GLO-1 protein after incubation with MG for 6, 24, and 48 h. (d) Quantified diagram showing the ratio of GLO-1/β-actin vs. incubation time for 0~48 h. Triplicate data were statistically treated, and results are expressed as the mean±SD (*n* = 3). Different levels of significance are expressed as * *p*<0.05 and *** *p*<0.001 compared to the control.

GLO-1 activity was also highly stimulated by MG treatment (**Fig 4A**). When treated with 500 μM MG, GLO-1 activity in NRK52E cells was immediately provoked within 30 min to 0.4 units/mg-protein, and its activity was respectively stimulated to 0.40, 0.44, 0.46 and 0.44 units/mg-protein at 1, 4, 6, and 24 h (*p*<0.01) compared to the initial activity of 0.32 units/mg-protein (**Fig 4A**). On the other hand, GLO-1 activity was also activated in a dose-dependent manner, reaching 0.24, 0.23, and 0.32 units/mg-protein when respectively treated with doses of 300 (*p*<0.05), 500 (*p*<0.05), and 700 μM (*p*<0.001) for 24 h (**Fig 4B**). MG upregulated GLO-1 expression to 121% compared to 100% of the control after NRK52E cells were treated with 500 μM MG for 48 h (**Fig 4C and 4D**).

### 3.5. MG stimulated reactive oxygen species (ROS) production and upregulated Nrf2 expression in NRK52E cells

**Fig 5:**
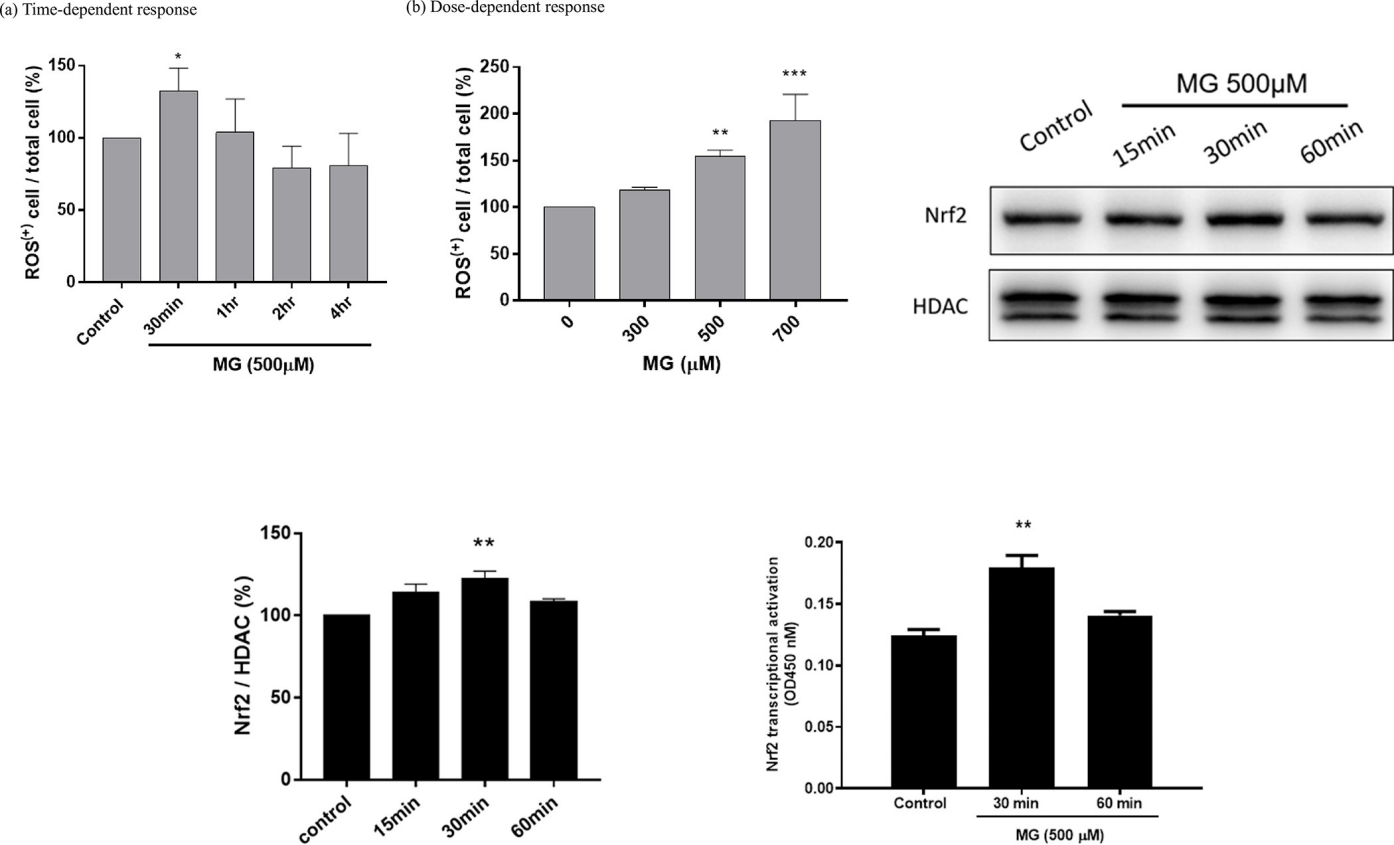
Time- and dose-dependent intracellular reactive oxidative stress responses and Nrf2 expression affected by MG. (a) Time-dependent reactive oxygen species (ROS) production per cell. NRK52E cells seeded onto six-well plates at a density of 1.5×10^6^ cells/well, were treated with MG (500 μM) for 0.5~4 h. Harvested cells were treated with fluorescent DHE dye and subjected to a flow cytometric fluorescence analysis. (b) Dose-dependent ROS production per cell treated with MG (0~700 μM) for 30 min. Triplicate data were statistically treated, and results are expressed as the mean±SD (*n* = 3). ROS levels were measured with a Muse^®^ Oxidative Stress Kit (Luminex). (c) Western blotting of Nrf2 from NRK52E cells treated with MG (500 μM) for 15, 30, and 60 min. (d) Quantified bar diagrams of data obtained from Western blotting of Nrf2 (*n* = 3). (e) The Nrf2 transcriptional activity in NRK52E cells treated with MG 500 μM at 30 and 60 min. Different levels of significance are expressed as *p<0.05, ** *p*<0.01, and *** *p*<0.001 compared to the control.

MG (500 μM) significantly stimulated the production of ROS in NRK52E cells to a peak of 135%±20% at 30 min, and then it decreased to 105%±27%, 78%±20%, and 79%±23% at 1, 2, and 4 h, respectively (**Fig 5A**). ROS production dose-dependently increased to 117%±4%, 155%±7%, and 190%±25% upon respective treatment with 300, 500 and 700 μM MG at 30 min (**Fig 5B**). In parallel, nuclear Nrf2 was significantly upregulated to 122%±5% at 30 min, and then decreased to 106%±2% by 60 min after being treated with 500 μM MG (**Fig 5C and 5D**). The Nrf2 activity was also similar to Nrf2 expression when cells were treated with MG 500 μM (**Fig 5E**).

### 3.6. Expressions of PI3K/AKT and AMP activated protein kinase (AMPK) were affected by MG

**Fig 6:**
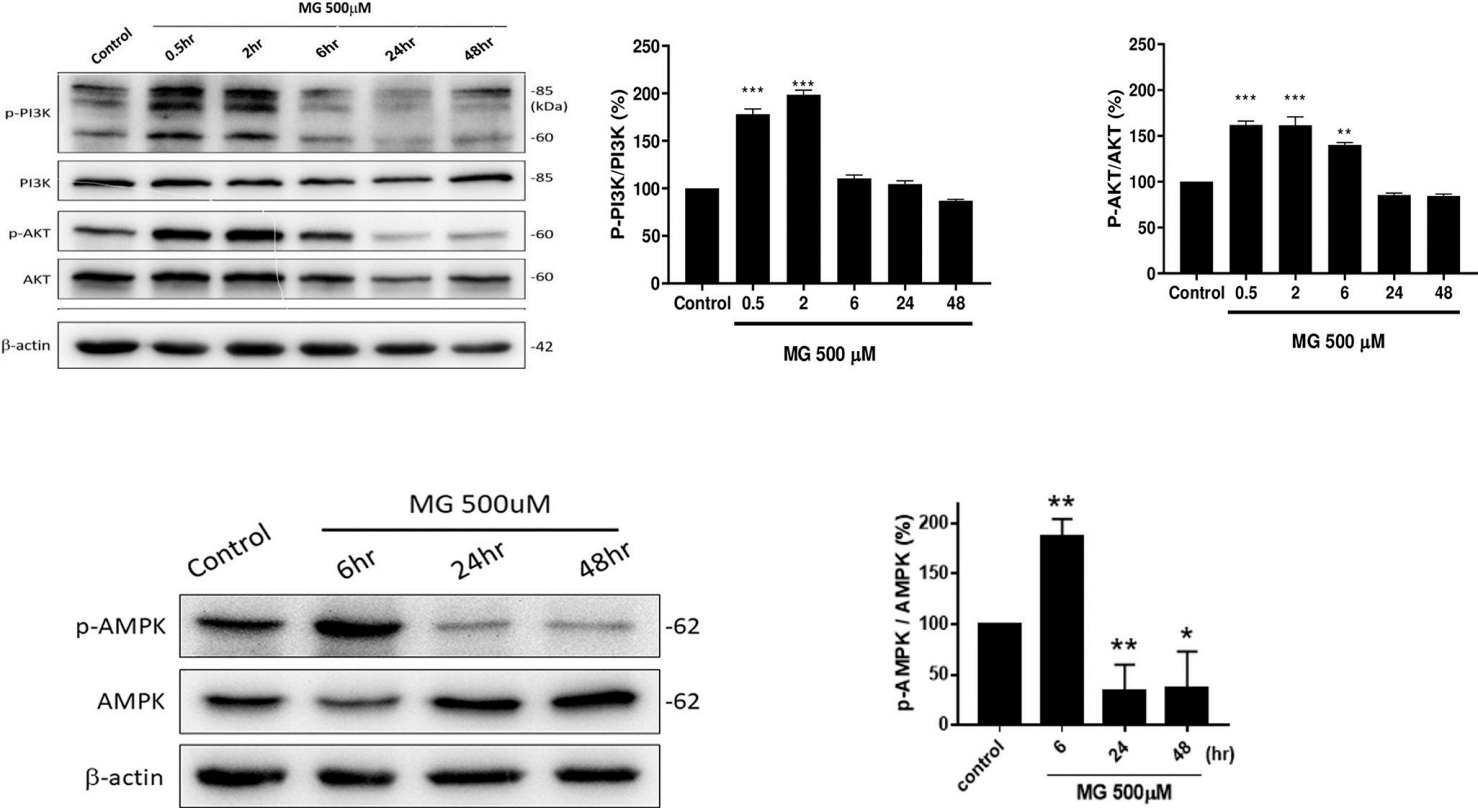
PI3K/Akt/AMPK pathways in NRK52E cells affected by MG. NRK52E cells, treated with MG (500 μM), were incubated for 0.5, 2, 6, 24, and 48 h. Cell lysates were collected and subjected to a Western blot analysis. (a) Western blotting of phosphorylated (p)-PI3K/PI3K, p-AKT/AKT, and an internal control protein (β-actin). (b) Quantified data of Western blot of p-PI3K/PI3K and p-AKT/AKT. (c) Western blotting of p-AMPK and AMPK. (d) Image of quantification of Western blot of p-AMPK/AMPK, with β-actin as an internal control. The image was quantified with ImagePro software. Data were statistically treated, and results are expressed as the mean±SD (*n* = 3). Different levels of significance are expressed as *p<0.05, **p<0.01, and ***p<0.001 compared to the control.

The PI3K/AKT pathway is one of the most well-known ROS-regulated pathways [13]. AMPK is required for Akt phosphorylation and activation under various cellular stresses [15]. After treatment with 500 μM MG, the ratio of p-PI3K/PI3K was stimulated at 0.5 and 2 h to 180% and 200%, respectively, and then declined to 110% at 6 h and to 86% at 48 h (**Fig 6A and 6B**). Similarly, the p-AKT/AKT ratio respectively increased to 162%, 161%, and 140% at 0.5, 2, and 6 h, then decreased to 86% at 24 h and 84% at 48 h (**Fig 6A and 6B**). p-AMPK was also highly expressed at 6 h (to 187%±17%) (*p*<0.001), and then declined through 24 h (32%±22%) (*p*<0.01) to 48 h (33%±20%) (*p*<0.05) (**Fig 6C and 6D**).

### 3.7. MG enhanced intracellular lipid accumulation in NRK52E cells

**Fig 7:**
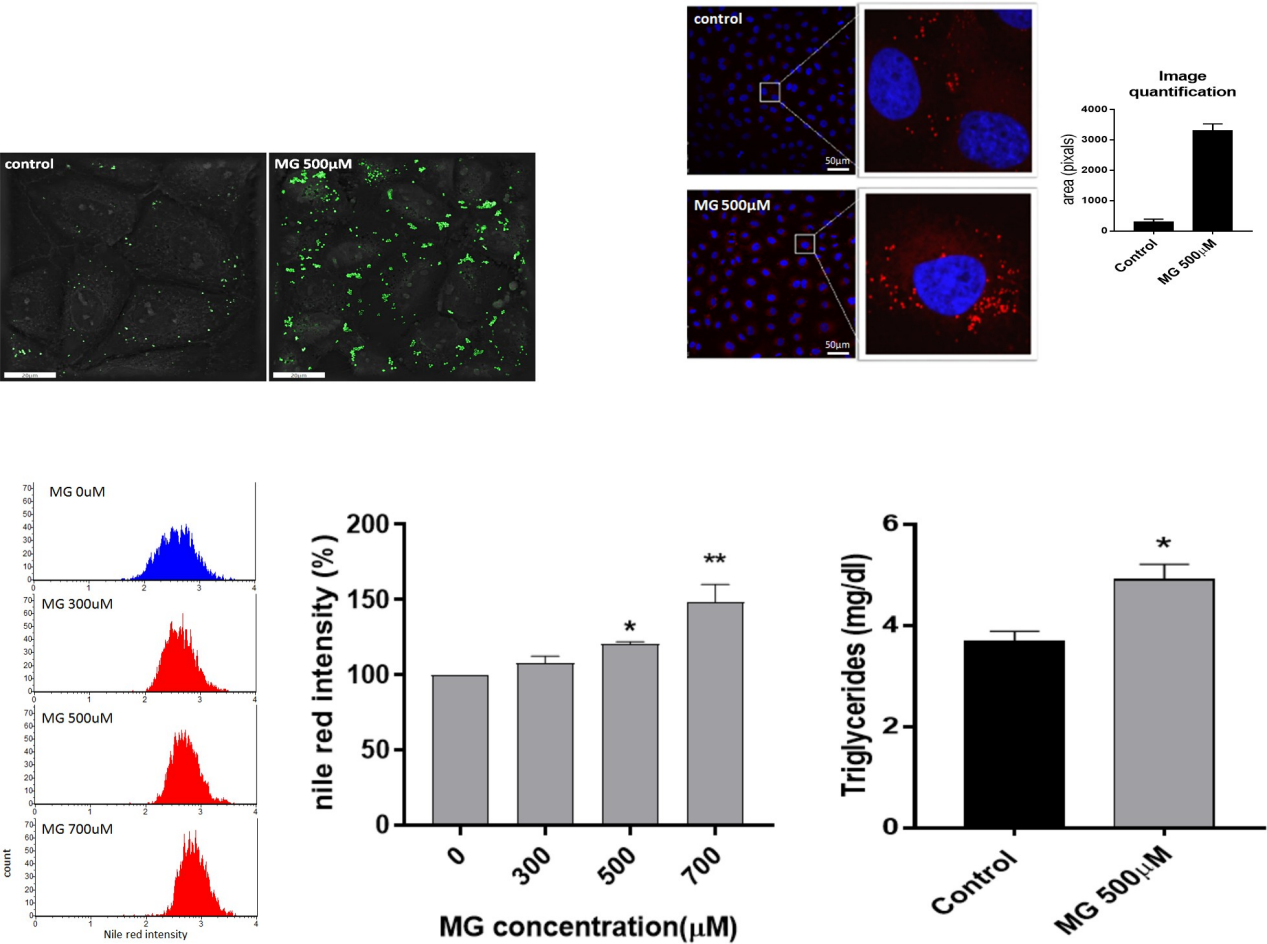
Intracellular lipid accumulation affected by MG. (a) Lipid droplet distribution under Nanolive 3D Cell Explorer. NRK52E cells, cultured in 35-mm μ-Dishes (from ibidi) were treated with MG (500 μM) for 24 h and subjected to Nanolive 3D Cell Explorer (Nanolive) and STEVE^®^ software analyses. Differential diffraction illustrates intracellular lipid droplet distribution. (b) Confocal microscopy of Nile red fluorescence with DAPI stain in NRK52E cells. Cells were treated with MG (500 μM) for 48 h, fixed, stained with Nile red and DAPI, and observed with an inverted microscope (magnification, 200x). Areas were integrated with Image Pro software, and results are expressed as the mean±SD (*n* = 10). (c) NRK52E cells were incubated with different dosages (0, 300, 500, and 700 μM) of MG for 48 h and subjected to a flow cytometric analysis at 2000 cells per event. Abscissa: the intensity of Nile red. Ordinate: the cell count. With higher intracellular lipid accumulation, Nile red signals further shifted to the right. d) Nile red flow cytometric data were statistically analyzed, and results are expressed as the mean±SD (*n* = 3). (e) NRK52E cells were treated with MG (500 μM) for 48 h. Harvested cells (10^7^ cells) were suspended in 500 μL dilution agent while being cooled on ice and subjected to ultrasonication at 5 s of ultrasonication/20 s stop per cycle for 10 cycles and centrifuged at 10^4^ *g* for 10 min, and the supernatant was separated and analyzed for triglyceride contents (mg/dL) with a Triglyceride Colorimetric Assay Kit. Data were statistically treated, and results are expressed as the mean±SD (*n* = 3). Different levels of significance are expressed as *p<0.05 and **p<0.01 compared to the control.

Lipid droplets were increasingly visible at 24 h after treatment with 500 μM MG (**Fig 7A**). As shown, the major fraction of lipid droplets exhibited sizes smaller than 5 μm, while some had sizes within 7~10 μm that contributed to the second major fraction (**Fig 7A**). Image quantification of Nile red staining by confocal microscopy showed that the orange-fluorescent area of cells treated with 500 μM of MG reached 3300±300 pixels compared to 250±50 pixels in the controls (**Fig 7B**).

The flow cytometric analysis of Nile red staining for 48 h further showed that the amount of intracellular lipid accumulation increased in a dose-dependent manner with the concentration of MG (**Fig 7C**). The fluorescent intensity (median on the abscissa) of Nile red staining shifted from 2.60 in the control to 2.65 at 300 μM, 2.70 at 500 μM, and 2.80 at 700 μM (**Fig 7C**), corresponding to 100% (at 0 μM MG) for the control, 105%±5% by 300 μM and 120%±3% by 500 μM (*p*<0.05), and to a higher intensity of 149%±15% by 700 μM (*p*<0.01) (**Fig 7D**). On the other hand, when treated with 500 μM MG, intracellular TGs are highly accumulated, reaching a concentration of 4.9±2.5 mg/dL compared to 3.6±2.0 mg/dL of the controls (*p*<0.05) (**Fig 7E**).

### 3.8. SREBPs and lipid metabolism-related proteins affected by MG

**Fig 8:**
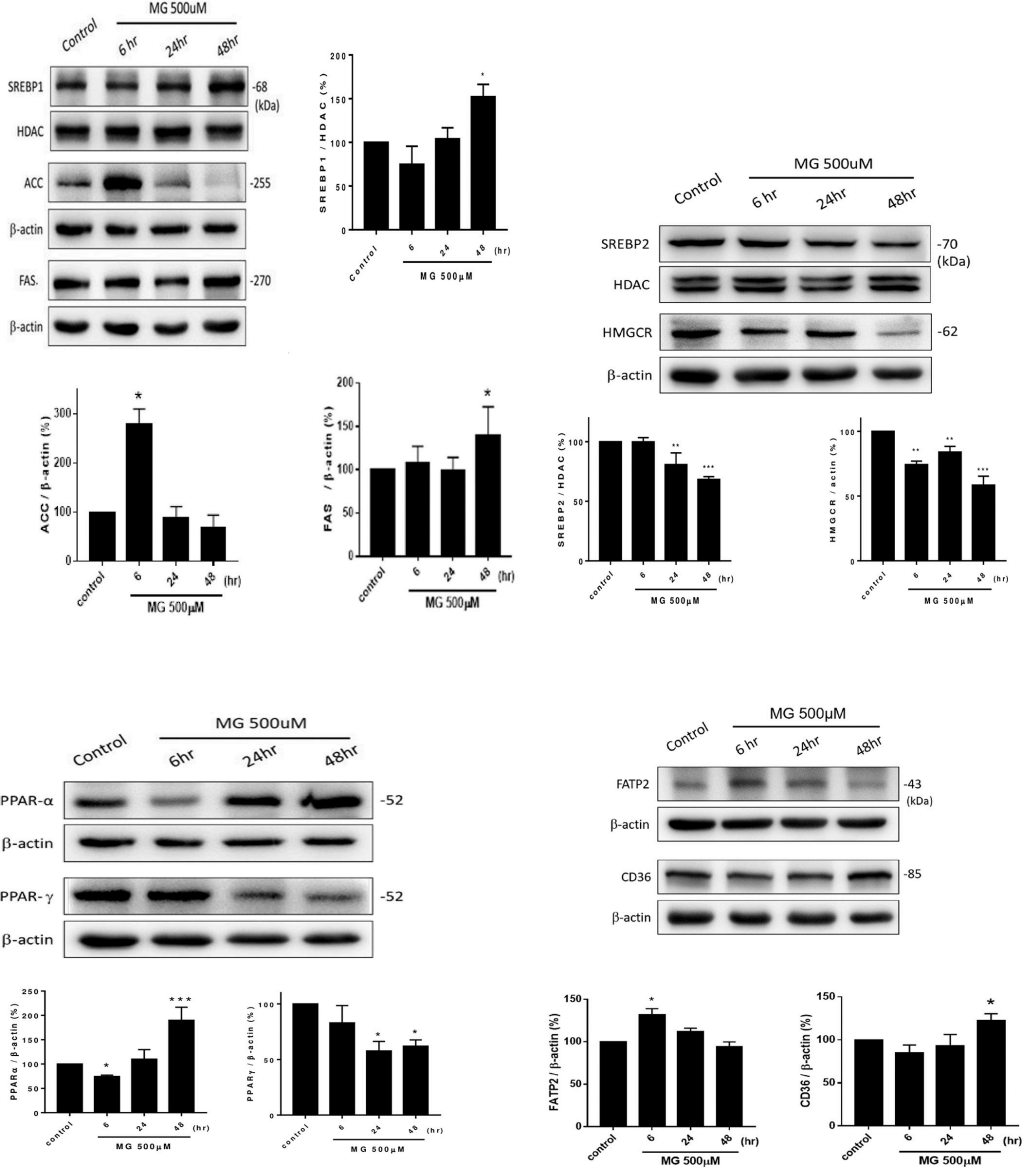
Lipid metabolism-related proteins affected by MG. NRK52E cells, treated with MG (500 μM), were incubated for 6, 24, and 48 h. Cell lysates were collected and subjected to a Western blot analysis. (a) Western blot pattern and image quantification of SREBP1, ACC, and FAS. (b) Western blot pattern and image quantification of SREBP2 and HMGCR. (c) Western blot pattern and image quantification of PPARα and PPARγ internal control protein. (d) Western blots indicating the protein expressions of FATP2, and CD36. Internal control proteins were HDAC for nuclei and β-actin for the cytosol. Images were quantified with ImagePro software. Data were statistically analyzed, and results are expressed as the mean±SD (*n* = 3). Different levels of significance are expressed as * *p*<0.05, ** *p*<0.01, and *** *p*<0.001 compared to the control.

When NRK52E cells were treated with 500 μM MG, mature SREBP1 was steadily upregulated to 102% and 151% (*p*<0.05) at 24 and 48 h, respectively (**Fig 8A**). In contrast, expression of mature SREBP2 was inhibited in a time-dependent manner from 100% at 6 h to 80% (*p*<0.01) and 70% (*p*<0.001) at 24 and 48 h, respectively (**Fig 8B**). ACC (Acetyl CoA-carboxylase) was momentarily activated to 280% at 6 h (*p*<0.05), and then returned to normal levels after 24 h (**Fig 8A**). FAS (270 kDa) initially remained unchanged until 24 h and then increased up to 140% at 48 h (**Fig 8A**). HMGCR was suppressed to 70% at 6 h, then to 82% and 56% at 24 and 48 h, respectively, compared to the controls (100%) (**Fig 8B**).

The PPARα level was downregulated at 6 h to 70%±2% (*p*<0.05) and then steadily increased to 108%±15% at 24 h and 180%±35% at 48 h (*p*<0.001). The PPARγ level was sequentially downregulated to 77%±20% at 6 h, 57%±8% at 24 h, and 60%±7% a 48 h (*p*<0.05) (**Fig 8C**) compared to the controls (100%).

Meanwhile, the FATP2, in NRK52E cells treated with MG (500 μM) responded within 6 h to 130% compared to the controls (100%) (*p*<0.05), and then time dependently declined. CD36 remained unchanged before 24 h and had increased to 122% by 48 h (**Fig 8D**).

### 3.9. The expression of FAS was modulated by Glo1 modulator on NRK52E

**Fig 9:**
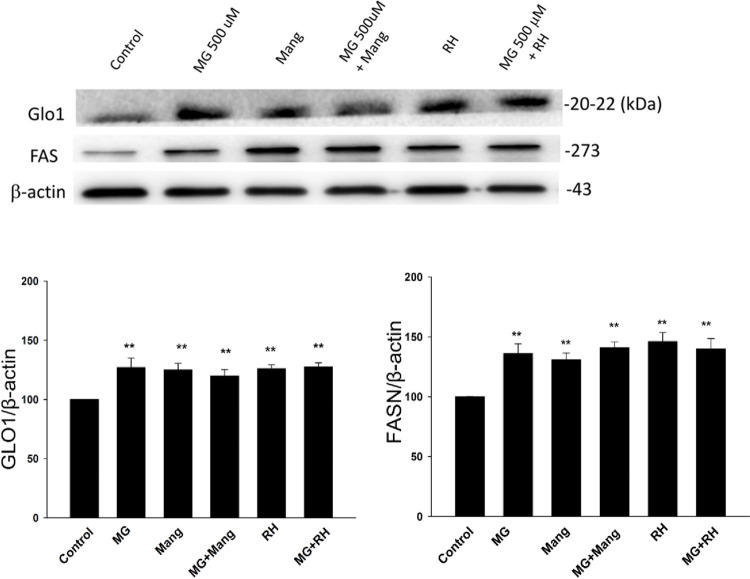
The expression of FAS was modulated by Glo1 inducers on NRK52E. NRK52E cells treated with Mang or RH with/without MG (500 μM) were incubated for 48 h. Cell lysates were collected and subjected to a Western blot analysis (a) and image quantification (b) for the Glo1 and FAS (**p<0.01 vs. control). Western blot analysis for the Glo1 and FAS (**p<0.01 vs. control).

Previously, mangiferin (Mang) and a combination of trans-resveratrol and hesperetin (RH) were suggested to be Glo1 inducers [9, 16]. To investigate whether Glo-1 modulation will affect the triglyceride accumulation in *de novo* fatty acid synthesis on NRK52E cells, we examined the effects of Mang, RH and BrBzGCp2 (Br, Glo1 inhibitor) separately on the protein expression of FAS in the NRK52E cells treated with MG (**Fig 9**). After the cells were cultured for 48 h, it was found that the protein expressions of Glo-1 were increased in both Mang and RH groups, but decreased in Br group significantly compared to the control group (*p* < 0.05). The level of FAS proteins were all significantly elevated as well as Glo1 upregulated (*p* < 0.05) but only decreased in cells cotreated with MG and Br.

## 4. Discussion

The accumulation of lipids in the kidney is known as renal lipotoxicity [17]. The majority of the fatty composition was TGs (**Figs 1C and 7E**) and not cholesterol or phospholipids, consistent with Lemieux et al. (1984) [6]. Excess lipid accumulation in non-adipose tissues may arise in the setting of high plasma free FAs or TGs determined by a mismatch between TG synthesis and breakdown, where *de novo* lipogenesis plays an important role in ectopic lipid accumulation [18]. Statistically, the increased prevalence of obesity parallels the increase in the incidence of CKD, implicating a close connection of lipotoxicity with the development of kidney diseases [17].

MG was reported to damage endothelial cells and play a role in developing hypertension, insulin resistance, and nephropathy [19]. Excess MG produced both by hyperglycemia and ROS blocks the sensing of adenosine monophosphate by AMPK [20], thereby favoring gluconeogenesis and lipogenesis in the liver [21]. Li et al. [22] also suggested that advanced oxidation proteins cause lipotoxicity and tubulointerstitial fibrosis of kidney.

When cellular DNA is damaged, two options adopted by damaged cells are either to stop the cell cycle or to eliminate the damaged cells by apoptosis [23]. Cyclin D1 exhibits a well-known growth stimulatory effect that regulates the G_1_ phase of the cell cycle by binding to and stimulating CDK4 and CDK6 activities [24]. Once DNA is damaged, cyclin D1 is degraded, and cyclin D-CDK4/6 complexes are disrupted, contributing to cell cycle arrest. However, even at this stage, a relatively small pool of cyclin D1 persists after damage and redistributes to the damaged DNA sites, leading to positive DNA damage repair. Nevertheless, such a forced overexpression of cyclin D1 might override cell cycle arrest, initiating DNA replication stress and, ultimately apoptosis [25].

In our finding, MG directly damaged NRK52E cells and disrupted the cyclin D1 and CDK4 at 6h, contributing to the halting of the G1 phase. However, following rapid upregulation of cyclin D1-CDK4 complex might be associated with the onset of apoptosis (**Fig 3A & 3B**). The procaspase 3/p21 complex formation is an essential system for cell death, caspase 3-mediated cleavage of p21 is involved in the induction of apoptosis [26]. The p21 increased progressively to peak level at 24h but decreased at 48h, which was in line with the activation of caspase 3 at 48h. An increase of p21 protected cell apoptosis at 24h and inhibited the elevation of cleaved caspase 3, but at 48h, when the caspase 3 increased to override the protective function of p21, caspase 3 mediated p21 cleavage, the expression of p21 was significantly attenuated (**Fig 3C**), cell apoptosis occurred.

Suggestively, upregulated cyclin D and CDK4 (**Fig 3**) could mean the adaptation of NRK52E cells to the MG insult. Similar reports described that CDK was activated during tubular epithelial hypertrophy in experimental diabetes, and initial tubular epithelial cell hypertrophy is considered “compensatory” and “adaptive” hypertrophy. However, over time, tubular cell hypertrophy becomes “maladaptive,” resulting in tubular atrophy and tubular-interstitial fibrosis, which are often endpoints of many renal diseases with diverse etiologies [27]. Jan et. al. suggested that MG-induced cell death involved apoptotic and necrotic events in canine renal tubular cells, due to the fact that MG induced a significant cell type-specific rise in intracellular Ca^2+^ concentration in renal tubular cells, and such strong Ca^2+^ signaling may play an important role in the early process of the cytotoxic action of MG [28]. Our finding of intense expression of caspase-3 (**Fig 3**) seemed to be well in line with most studies identifying apoptosis as the predominant mode by which MG causes cell death [29].

The GLO-1 system, a glutathione (GSH)-dependent enzymatic pathway, is the rate-limiting step in MG degradation, which makes its regulation tightly associated with MG toxicity [30]. An abnormal increase in MG dicarbonyl stress is a representative marker of diabetic kidney disease, which induces downregulation of renal GLO-1 and increasing flux of MG-derived hydroimidazolone (MG-H1) formation [10]. Protein inactivation and dysfunction linked to the dicarbonyl proteome may accelerate CKD development [10] Higher production of MG consumes more GSH, and eventually, a depleted status of GSH may occur, resulting in an imbalance of the *in vivo* oxido-reduction status [31] ROS may upregulate profibrotic molecules such as transforming growth factor-beta 1 and plasminogen activator inhibitor-1 [17]. From the results of this study, GLO-1 activity first increased after 30 min in response to MG stimulation to eliminate toxicity (**Fig 4**). However, cellular damage may persist due to insufficient GSH under oxidative stress to relieve dicarbonyl stress. It is worth noting that GLO-1 was also found to be frequently overexpressed in various types of cancer, which may reflect the fact that cells grow with high glycolytic activity and high flux of MG formation [11]

Nrf2 mediates the transcriptional response of cells to oxidative stress. As a short-lived transcription factor, Nrf2 is active only when it is translocated into nuclei, and translocated Nrf2 is largely regulated by its subcellular distribution [32]. In CKD, oxidative stress is partly due to a diminished antioxidant capacity which is largely caused by impairment of Nrf2 activation [33]. The expression pattern of Nrf2 (**Fig 5D & 5E**) coincided well with ROS production (**Fig 5A**), suggesting speculated transient translocation of upregulated Nrf2 into nuclei, which is responsible for signaling by other downstream protective gene expressions [32]. AMPK and Nrf2 signaling may cooperate in readjusting cellular homeostasis [34]. PI3K/AKT pathway signaling plays an important role in modulating Nrf2 signaling [13]. AMPK and AKT are two primary effectors in response to metabolic stress. AMPK acts as an energy-sensing factor which rewires metabolism and maintains a redox balance [15]. Consistent with the appearance of peak ROS at 30 min after MG treatment (**Fig 5A**), upregulation of p-PI3K/PI3K and p-AKT/AKT also appeared at the highest levels from 0.5 to 2 h (**Fig 6B**). This pathway has been most extensively characterized in regulating apoptosis and cellular proliferation [13] (**Fig 2**). Similarly, the p-AMPK/AMPK ratio first increased at 24 h and then decreased at 48 h. As found in parallel with ROS production (**Fig 6A**) and p-PI3K and p-AKT upregulation (**Fig 6B**), profound expression of Nrf2 also reached a peak at 30 min (**Fig 5D & 5E**). Acting as an energy-sensing factor, AMPK is activated when cells are insulted with MG [35]. The immediate AMPK activation of NRK52E cells after MG treatment also seemed to reflect that cells struggled to protect themselves from MG-induced apoptosis [36].

In addition to coordinating an antioxidant response to oxidative stress, there is a potential role of Nrf2 in regulating metabolic processes, including nicotinamide adenine dinucleotide phosphate (NADPH) production and the metabolism of lipids, amino acids, and nucleotides [34]. The progressive decrease in Nrf2 was correlated with an increase in lipid accumulation as lipid droplets after cells were treated with 500 μM MG (**Figs 5C–5E and 7**). Nrf2 is a negative lipid level regulator [37]. Huang et al. suggested Nrf2 may play a role in modulating lipid homeostasis through transcriptional activation of a small heterodimer partner and lipogenic gene expressions [38]. Nrf2 inhibited lipid accumulation and oxidative stress in the mouse liver after HFD feeding, probably by interfering with lipogenic and cholesterologenic pathways [37]. More recently, Chen et al. demonstrated that Nrf2 can regulate the expression of long-chain acyl-CoA synthetase-1 (ACSL1), which plays a direct role in renal lipid deposition [39]. Therefore, we also determined the proteins of lipid metabolism pathways of NRK52E cells treated with MG (**Fig 8**).

SREBP-1 primarily enhances transcription of genes required for FA synthesis, while SREBP-2 preferentially activates cholesterol synthesis [40]. Upregulation of SREBP-1 induces TG accumulation (**Figs 7E & 8A**). Proteolytic processing and nuclear translocation of SREBP-1 are inhibited by AMPK, conversely, PI3K/AKT activates SREBP1 [41]. However, a bidirectional relationship could also exist between AKT and SREBPs, whereby an overactive AKT enhances SREBP activity and lipid accumulation, which in turn upregulates AKT signaling [42] (**Figs 6 and 8**).

PPARα, responsible for lipid metabolism, enhances the uptake, storage, and catabolism (*via* increasing β-oxidation) of free FAs [43]. Impairment of PPARα leads to lipid disorders, e.g., lipotoxicity [44]. In contrast, PPARγ, closely related to glucose metabolism, inhibits the uptake, storage, and oxidation of free FAs but stimulates insulin sensitivity and glucose utilization in skeletal muscles [43]. Suppression of PPARγ induces diabetes implicating an adverse effect of MG of enhancing DM severity (**Fig 8C**) [44]. A previous study reported that FAS cooperates with GLO-1 to protect against sugar toxicity [45]. Taken together, the simultaneous upregulation of p-PI3K/p-AKT, p-AMPK, PPARγ, ACC, FAS, and SREBP1 and downregulation of PPARα, HMGCR, and SREBP2 (**Figs 6 and 8**) may lead to overriding protection and a huge deposition of TG-rich lipid droplets (**Fig 7**) in renal tubes. Herman-Edelstein et al. indicated that there were highly significant correlations among the glomerular filtration rate, inflammation, and lipid metabolism genes, evidencing the possible role of abnormal lipid metabolism in the pathogenesis of DM [46]. Downregulation of SREBP2 and HMGCR reduced cholesterol biosynthesis, which in turn facilitated FA biosynthesis from the common precursor, acetyl CoA.

FATPs, also known as solute carrier protein family 27 (SLC27), include six proteins that are associated with the transport of long-chain FAs (LCFAs) in the plasma and across intracellular membranes and function as a gate in the regulated transport of FAs [47]. The uptake of LCFAs plays a pivotal role in metabolic homeostasis and human physiology [48, 49] FATPs are direct PPARα target genes, and deletion of SLC27 was shown to restore FA oxidation and protect renal tubule cells from lipotoxicity [50], which is implicated in our results. PPARα was downregulated, and FATP2 was upregulated at 6 h (**Fig 8**). CD36 and also LCFA transporters are involved in CKD development and have important roles in regulating FA oxidation and esterification, lipid accumulation, metabolic dysfunction, and numerous other functions [51]. In addition to changes in FA uptake, abnormalities of fat metabolism and the associated pathology might involve dysfunction of CD36-mediated signal transduction [51] Li et al. also reported high glucose-stimulated advanced oxidation protein product-induced lipotoxicity, apoptosis, and fibrosis *via* the CD36 receptor [22]. Excessive lipid accumulation in non-adipose tissues may accommodate the setting of high plasma free FAs or TGs. Although intracellular lipid metabolism is mostly regulated by FA synthesis and oxidation, FA transporters are also important modulators of lipid homeostasis. Excess free FAs may impair normal cell signaling and cause cellular dysfunction. In some circumstances, excess free FAs may induce apoptotic cell death [52]. Upregulation of FATP2 and CD36 may be involved in the pathogenesis of kidney fibrosis by abnormal FA uptake induced by MG in NRK52E cells (**Fig 8D**).

For in parallel clarifying the potential application and mechanism for Glo1 inducers on lipid accumulation induced by MG, we used natural compounds Mang and RH to check how FAS proteins affected when treated alone or cotreatment with MG on NRK52E cells. The results indicated the induction of Glo1 are involved in *de novo* lipogenesis-related upregulation of FAS (**Fig 9**). The correlation between Glo1 and FAS is likely to be the adaption of renal tubule cells damaged by dicarbonyl stress [45].

## 5. Conclusions

**Fig 10:**
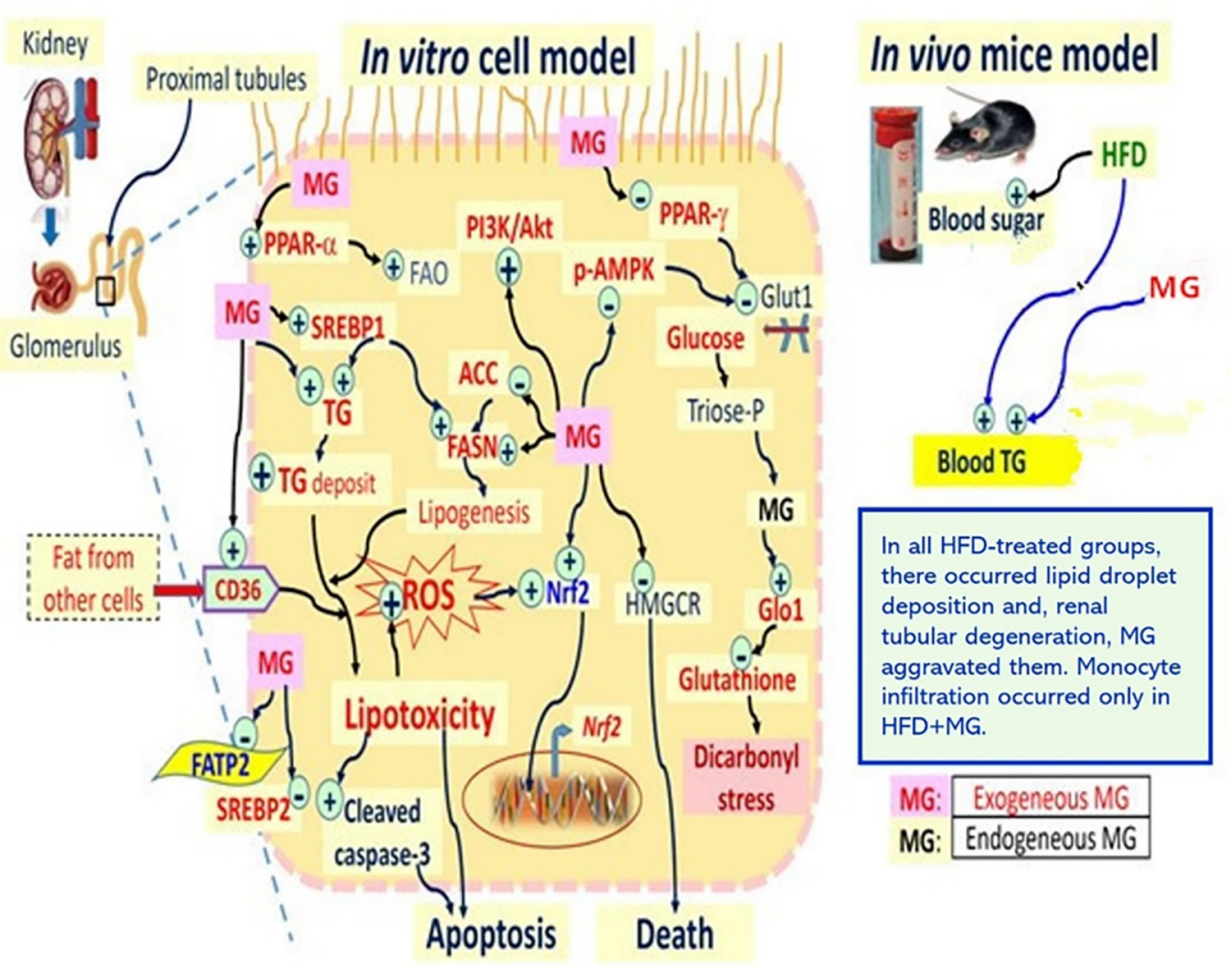
Graphic summary of potential signaling pathway involved in MG-induced lipotoxicity *in vitro* and *in vivo*. (FAS, fatty acid synthase; ACC, acetyl-CoA carboxylase; HFD, high-fat diet; FATP2, fatty acid transport protein 2; HMGCR, HMG-CoA reductase).

To summarize, DM elicits glucotoxicity, and is characteristically associated with dicarbonyl stress, which produces huge amounts of MG and glyoxal. In this study, from the NRK52E cell model which has been validated by many experimental animal results, we found that MG induced renal lipotoxicity in an *in vitro* cell model. MG activated the Nrf2/PI3K/AKT pathway and the downstream AMPK/SREBP pathway. Activated SREBP1 accompanied by downregulated SREBP2 stimulated ACC and FAS, but not HMGCR, implying that lipid accumulation and lipid droplet clustering in NRK52E cells were mostly contributed by free FAs and TGs, with only a small portion from cholesterol biosynthesis. Intracellular lipid droplet accumulation intensively increased ROS-bearing cells, accompanied by high Nrf2 expression. Meanwhile, PPARα was upregulated, while PPARγ was downregulated. PPARα elevates mitochondrial and β-oxidation rates of peroxisomal FAs, providing energy for peripheral tissues and playing a potential role in oxidant/antioxidant pathways. PPARγ acts as a sensor of hormones, vitamins, endogenous metabolites, and xenobiotic compounds. Nuclear receptors control expressions of a huge number of genes, causing insulin sensitization and enhancing glucose metabolism. In addition, the cell cycle-related proteins, cyclin D, CDK4, and cleaved caspase-3, were all time-dependently activated by MG, evidencing G_0_/G_1_ phase stop associated with simultaneous apoptosis of NRK52E cells. A graphic summary is shown in **Fig 10**.

An abnormal increase in MG dicarbonyl stress is a characteristic of CKD. Protein inactivation and dysfunction linked to the dicarbonyl proteome contribute to CKD development, while the accumulation of lipids in the kidneys was recently referred to as renal lipotoxicity. Current knowledge on renal lipid physiology and pathophysiology indeed is still insufficient, and our findings together with others may provide a strong foundation and incentive for further exploration.

## Supporting information

S1 Raw images The original uncropped and unadjusted images relating to Figs 3A, 3C, 4C, 5C, 6A, 6C, 8A-8D and 9. (RAR)
